# Dietary protein, amino acids and type 2 diabetes mellitus: a short review

**DOI:** 10.3389/fnut.2024.1445981

**Published:** 2024-07-24

**Authors:** Marco Mensink

**Affiliations:** Division of Human Nutrition and Health, Chair Group Nutritional Biology, Wageningen University & Research, Wageningen, Netherlands

**Keywords:** diabetes, dietary protein, insulin resistance, branched chain amino acids, high-protein

## Abstract

Diabetes is a widespread metabolic disorder and results from insulin resistance and impaired insulin secretion. Modifiable factors like diet, physical activity, and body weight play crucial roles in diabetes prevention, with targeted interventions reducing diabetes risk by about 60%. High-protein consumption, above the recommended intake of 0.8 g/kg body weight per day, have often explored in relation to diabetes risk. However, the relationship between dietary protein and diabetes is multifaceted. Observational studies have linked high total and animal protein intake to an increased risk of type 2 diabetes, particularly in obese women. Elevated levels of branched-chain amino acids (BCAA), which can result from dietary intake, protein breakdown, as well as an impaired catabolism, are strong predictors of cardiometabolic risk and insulin resistance. With several mechanism linking BCAA to insulin resistance. On the other hand, intervention studies suggest that high-protein diets can support weight loss and improve cardiometabolic risk factors. However, the impact on insulin sensitivity and glucose homeostasis is not straightforward. Proteins and amino acids stimulate both insulin and glucagon secretion, influencing glucose levels, but chronic effects remain uncertain. This short narrative review aims to provide an update on the relationship between increased dietary protein intake, amino acids, insulin resistance and type 2 diabetes, and to describe protein recommendations for type 2 diabetes.

## Introduction

Diabetes is a prevalent metabolic disorder worldwide, characterized by elevated blood glucose levels. Its prevalence has been steadily increasing, with an estimated 537 million adults aged 20–79 living with diabetes globally in 2021, and a further rise expected (1). Type 2 diabetes comprises the majority of cases, accounting for around 90% of all diabetes cases. Disruptions in insulin action and secretion contribute to the characteristic hyperglycaemia (2). Cells become resistant to insulin’s actions, most notably insulin-resistant glucose uptake in skeletal muscle which results in elevated blood glucose levels. Initially, the pancreas may produce extra insulin to compensate for insulin resistance. However, over time, the pancreatic beta cells may fail, leading to decreased insulin production and exacerbating high blood glucose levels. Both the hyperglycaemia as well as the chronic exposure to elevated insulin levels can in their turn further diminish insulin-mediated glucose uptake, potentiating insulin resistance and eventual development of type 2 diabetes mellitus.

Diet, physical activity and body weight are key modifiable factors for the development of diabetes. Targeted interventions aiming at these three factors have shown to reduced diabetes risk by ~60% in those at risk for the disease (3–5). Various diets aimed at weight reduction and improving insulin sensitivity are advocated for patients with diabetes, among them high-protein diets. With high protein diets or high protein consumption being defined as a protein intakes above the general recommended level of 0.8 g/kg BW /per day for healthy adults (6). Already a century ago dietary protein intake was investigating in relation glycemic control (7), due to its glucogenic properties as well as its stimulation of insulin and glucagon secretion (8). In addition, high-protein diets are recommended for weight-loss and maintenance (9), which might benefit diabetes risk reduction. Also, a –too—low protein intake, for example as part of protein-energy malnutrition in older adults, is associated with morbidity and mortality (10). However, the impact of high-protein diets or high protein consumption on insulin sensitivity and – risk of – diabetes is not straightforward, as we reviewed 10 years ago (11). Observational studies have identified a high protein intake as a risk factor for type 2 diabetes mellitus, and elevated circulating branched-chain amino acids (BCAA) levels are among the strongest predictors of future cardiometabolic risk. Also, some amino acids can have a direct impact on hormones and pathways that control glucose homeostasis.

This short narrative review aims to provide an update on the relationship between increased dietary protein intake, amino acids, insulin resistance and type 2 diabetes, and to describe protein recommendations for type 2 diabetes.

### Population studies: high-protein diets are associated with type 2 diabetes

Besides calorie intake, fiber and carbohydrate consumption, also protein intake has been investigated in relation to diabetes risk in large scale observational studies. Within the setting of EPIC-InterAct, a large scale pan-European type 2 diabetes case-cohort, we were able to study the association between protein intake and risk of type 2 diabetes (12). After adjustment for important diabetes risk factors and dietary factors, the incidence of type 2 diabetes was higher in those with high intake of total protein and animal protein, not plant protein (12). Associations were stronger in women, more specifically obese women. A meta-analysis of several large population studies, including the large Nurses’ Health Studies and the Melbourne collaborative cohort study confirmed these observations: higher intakes of total and animal protein were both associated with increased risks of T2D, whereas higher plant protein intake tended to be associated with lower risk of T2D (13). High (animal) protein intake is in ‘free-living’ conditions in general related to a higher (saturated) fat intake, a lower fiber and vitamin intake, increased BMI and lower physical activity levels (14). These are known diabetes risk factors and could contribute to the positive association between protein intake and diabetes risk. Indeed, including these – and other – factors into subsequent models attenuated the association, but the positive association between total or animal protein and diabetes risk did not disappear (12, 13).

In particular the BCAA attracted attention, as several studies identified high plasma levels of BCAA predicting diabetes risk (15, 16), and decreased levels were associated with improvement in insulin resistance (17). BCAA, which include leucine, isoleucine and valine, are all essential amino acids with a relative high presence in various protein sources, in particular those from animal origin. BCAA have, next to protein synthesis, multiple important roles in human metabolism and several metabolic diseases. Although acute infusion of BCAA can introduce insulin resistance (18), plasma BCAA levels are not simply a consequence of a high (animal) protein intake. Elevated circulating BCAA levels can have multiple origins, including increased appearance in plasma due to food intake, protein breakdown and gut microbial synthesis, and/or alteration in disappearance due to protein synthesis, excretion and BCAA catabolism. With the latter, a dysfunctional—repressed—BCAA catabolism has been proposed as playing a large role (19). Thus the relationship between BCAA, insulin resistance and type 2 diabetes is much more complex, and characterised as a “two-way street” (20), with diabetes, obesity and insulin resistance contributing to elevated BCAA levels and vice versa. Several strategies are considered to lower BCAA levels and/or boost BCAA catabolism, which includes diet and exercise interventions, next to pharmaceutical approaches (19). Interestingly, combining a high-protein diet with a high fiber intake diminished the correlations of AA with IR (21), which may be related to the effect of fiber on digestion and absorption of dietary protein.

Thus, observational data clearly identified a high (animal) protein intake under ‘free living’ non-restricted conditions to be associated with an increased risk of developing diabetes, with elevated circulating BCAA levels as biomarkers of disease risk. However, these associations do not immediately identify high protein consumption as a cause of diabetes, due to multiple other – dietary – factors being associated with a high protein consumption, and elevated circulating BCAA levels.

### Intervention studies: protein intake to support weight-loss and improved metabolic control

Protein intake is recommended to support weight-loss and weight maintenance, in particular to preserve lean mass when on calorie restriction. It is estimated that high protein intake (e.g., >1.0 g/kgBW/day) compared to normal protein intake (0.8 g/kgBW/day) can prevent a loss of 0.5–1.0 kg lean mass with moderate weight-loss (22, 23). Adequate protein intake stimulates muscle protein synthesis and hence supports lean mass. In addition, dietary protein is more satiating than fat or carbohydrate, and dietary protein stimulates thermogenesis, both also facilitating weight loss and maintenance. Interestingly results from the POUNDS LOST study suggest that optimal diet composition for weight loss depends on metabolic state of an individual. Those with normoglycemia lost the most body weight on a low-fat/high-protein diet, while subjects with insulin resistance lost the most on a high-fat/high-protein diet, most likely due to difference in the satiating effects of carbohydrates (24).

As body weight, and body weight-loss, is a key factor in insulin resistance and glucose homeostasis, high-protein diets may improve cardiometabolic risk factors. A recent systematic review and meta- analysis of 54 randomised controlled trials in populations without diabetes confirmed the impact of high protein diets (i.e., 20–45 Energy%) versus low-protein diets (i.e., 10-23E%) on weight-loss and fat mass loss (25). But also systolic blood pressure, total cholesterol, triacylglycerol and fasting insulin levels, a marker of insulin resistance, were lower on HP diets. No significant differences were seen for glucose, HbA1c and insulin resistance as estimated by HOMA-IR, although fewer studies assessed these effects (25). In patients with diabetes, a high-protein, low carbohydrate weight-maintaining diet improved fasting plasma and 24-h glucose and HbA1c level (26). Although protein is known for its effect on insulin (and glucagon) secretion by the pancreas, the low glucose availability is probably the key in the impact of this diet. In a moderate weight-loss trial in obese women, a high-protein (1.2 g/kg/day) regular carbohydrate diet compared to a low protein (0.8 g/kg/day) diet reduced the WL-induced decline in lean tissue mass, but it also prevented the WL-induced improvements in muscle insulin signalling and insulin-stimulated glucose uptake (23). Thus without a significant decrease in CHO intake the effect of weight-loss was blunted by a high protein intake.

Next, to total protein intake also protein source could play a role. In a randomised cross-over trial we compared two weight-maintenance high-protein diets (22En%) with different protein sources (27). Substituting 30 grams of protein daily from meat products with soy products in postmenopausal abdominally obese women led to improvements in various cardiometabolic risk factor, including insulin sensitivity as measured with an frequently sampled intravenous glucose tolerance test (FSIGT). Whether the protein itself explains these findings or the isoflavones associated with soy protein could not be concluded. From a systematic review on the effects of plant protein versus animal protein in healthy humans and those with a metabolic impairment, it was concluded that there is some evidence that the intake of plant protein, in particular soy protein associated with isoflavones, may prevent the onset of cardiometabolic risk factors like hypercholesterolemia and hypertension, but an effect on glucose homeostasis could not be concluded (28). From studies with individuals with diabetes, it was concluded that replacing sources of animal with plant protein leads to modest improvements in glycaemic control (29) Again, an important note is that changing plant-protein intake is in general part of changes in plant-based foods consumption with subsequent changes in other nutrients and dietary factors, like fiber, fat, micronutrients and energy. Attributing effects to (plant) proteins perse should therefore always be done careful.

Altogether, in controlled intervention studies, high-protein (energy restricted) diets improve body weight and composition as well as multiple cardiometabolic risk factors including insulin resistance and glycaemic control. High plant protein intake, as part of a plant-based diet, may have stronger effects compared to animal protein, as part of an animal-based diet, but mainly on lipids, not on glucose homeostasis. With other dietary factors than plant protein, like fat, fiber and micronutrient intake explaining the beneficial effect on in particular LDL cholesterol (30). In addition, a reduced glycaemic load associated with a higher protein intake may be an important factor explaining the effect on glucose metabolism.

### Protein, AA, and insulin and glucagon secretion

Protein and AA are known to stimulate insulin and glucagon secretion from the pancreas (8, 31). Insulin stimulates peripheral glucose uptake, in particular in muscle tissue, and hence can lower glucose levels. Indeed, when co-ingested with glucose protein and AA can stimulate insulin secretion, and can attenuate the glucose response, although these effects are relatively small in young healthy adults (8). The metabolic effects differ per AA, with isoleucine and phenylalanine resulting in the largest attenuation of glucose levels while leucine had the largest impact on insulin secretion when co-ingested with glucose (8). In type 2 diabetes, where insulin secretion after carbohydrate ingestion is severely impaired, amino acid and protein co-ingestion were shown to substantially increase plasma insulin responses (32), and can assist in acute metabolic control. Whether this acute stimulatory effect of AA on insulin is conserved over time, as well as whether this is desirable knowing the effect of chronic hyperinsulinemia on worsening of insulin resistance, needs to be established. Next to stimulation of insulin secretion, AA are also known to induce a rise in glucagon and to attenuate the glucose lowering effect of glucagon response (8), with again a different effect for different AA. Glucagon has multiple metabolic effects. Glucagon opposes insulin and stimulates gluconeogenesis and hepatic glucose output, resulting in maintenance or elevation of plasma glucose levels and availability for peripheral tissues. Elevated fasting glucagon levels (hyperglucagonemia) are present in obese individuals with (pre-)diabetes and are predictive of future diabetes development (33). The effect of protein and AA on glucagon secretion and circulating glucagon could be one of the mechanism underlying the observation of an increased diabetes risk associated with a high (animal) protein consumption (33). But glucagon also stimulates insulin secretion which in the prandial state could assist in glycaemic control (34). In addition glucagon activated hepatic lipolysis which lower hepatic lipids, a condition known to be associated with insulin resistance (35). Both insulin and glucagon responses are modulated by incretin responses, i.e., gastric inhibitory polypeptide (GIP) and glucagon-like peptide-1 (GLP-1). Nutrients as well as mixed meals trigger incretins, but the endogenous incretins do not seem to play a major role in the hyper glucagon secretion seen after a mixed meal in type 2 diabetes (36). It is proposed that targeted combinations of AAs that maximise insulin secretion and mitigate (fasting) hyperglucagonaemia, could be considered for clinical applications in patients with diabetes (31).

### BCAA, mTOR, and mitochondrial function

Elevated blood BCAA levels have bene identified as predictor of diabetes risk, and direct infusion of BCAA induces rapidly insulin resistance (18). Elevated BCAA levels are not just a simple reflection of a high protein intake. Next to food intake, also protein breakdown in tissue, a process which is inhibited by insulin, and gut microbial synthesis contribute to BCAA appearance and levels in the blood. Disappearance of BCAA on the other side is a consequence of protein synthesis, excretion and BCAA catabolism. All these processes together define the levels of BCAA in plasma, and in particular a dysfunctional or impaired breakdown of BCAA is thought to be an important factor in the elevated circulating BCAA levels in patients with (pre)diabetes (19). BCAA, and potentially toxic metabolites such as BCAA-derived acylcarnitines, can however directly or indirectly interfere with insulin action and contribute to the development of insulin resistance and diabetes (19, 20, 37).

A direct effect could be the persistent activation of the mTOR pathway. The mTOR pathway is a nutrient-sensing pathway, which integrates nutrient sensing and insulin signalling to coordinate cell growth and metabolism and it could have a crucial role in understanding the association between BCAA and insulin action (11). BCAA and other nutrients activate mTOR, which is well-known for its role in dietary protein stimulated (muscle) protein synthesis. Among other signals, this activation can also lead, in a negative feedback loop, to phosphorylation of the insulin receptor substrate 1 (IRS1), leading to a decreased insulin action (37).

Indirectly, BCAA, but in particular its potential toxic metabolites, may result in an impaired mitochondrial function, with reduced oxidation of lipid substrates resulting in accumulation of lipid in (muscle) cells (19, 20, 37). Lipid accumulation in muscle tissue is associated with insulin resistance, as lipid intermediates can interfere with insulin signalling, known as ‘lipotoxicity’, resulting in a reduced insulin stimulated glucose uptake and impaired glucose homeostasis (38). But lipotoxicity affects also normal function of other tissues, including the pancreatic (B-cell) and cardiac tissue.

### Protein recommendations for adults with – an increased risk of – type 2 diabetes

Several national bodies have in recent years released guidelines or statements for those with diabetes are at risk for developing diabetes (‘prediabetes’), including the American Diabetes association, ADA (39), Diabetes Canada (40), Diabetes UK (41) as well as the Dutch diabetes Federation, NDF (42). General consensus is that diabetes can be delayed or prevented by a healthy diet and increased physical activity, accompanied by weight loss. Dietary protein intake is not a key target in nutritional guidelines for patients with diabetes across the different countries. These dietary guidelines, supported by strong evidence, recommend restricting energy intake and weight-loss, increasing fiber intake, including low glycaemic index foods, and reducing saturated fat intake.

According to Nutrition Therapy guidelines of Canada Diabetes Association (40), there is no evidence that the usual protein intake for most individuals (1 to 1.5 g per kg body weight per day), representing 15 to 20% of total energy intake, needs to be modified for people with diabetes. This level of intake, that is generally observed in western countries, can already considered to be high-protein compared to the 0.8 g/kg BW recommendation of the FAO, although experts recommend an intake of ~1.2 gram/kg BW for older adults (43). Importantly, this intake in grams per kg per day should be maintained or increased with energy-reduced diets to maintain lean mass during weight-loss. As reviewed above, protein quality could be an another consideration, as replacement of animal protein with sources of plant protein could improve A1C, FPG and fasting insulin (29).

Finally, some words of caution are made for diabetes patients with chronic kidney disease (CKD) and when using a low-protein diet. In CKD patients a level of intake at 0.8 g/kg ideal BW is advised (40, 42), as protein restriction reduces end stage renal disease. However, harm due to protein malnutrition, in particular in older adults, should not be ignored (43), and quantity and quality of protein intake must be optimized at the individual levels to meet requirements for essential amino acids.

## Concluding remarks

**Figure 1:**
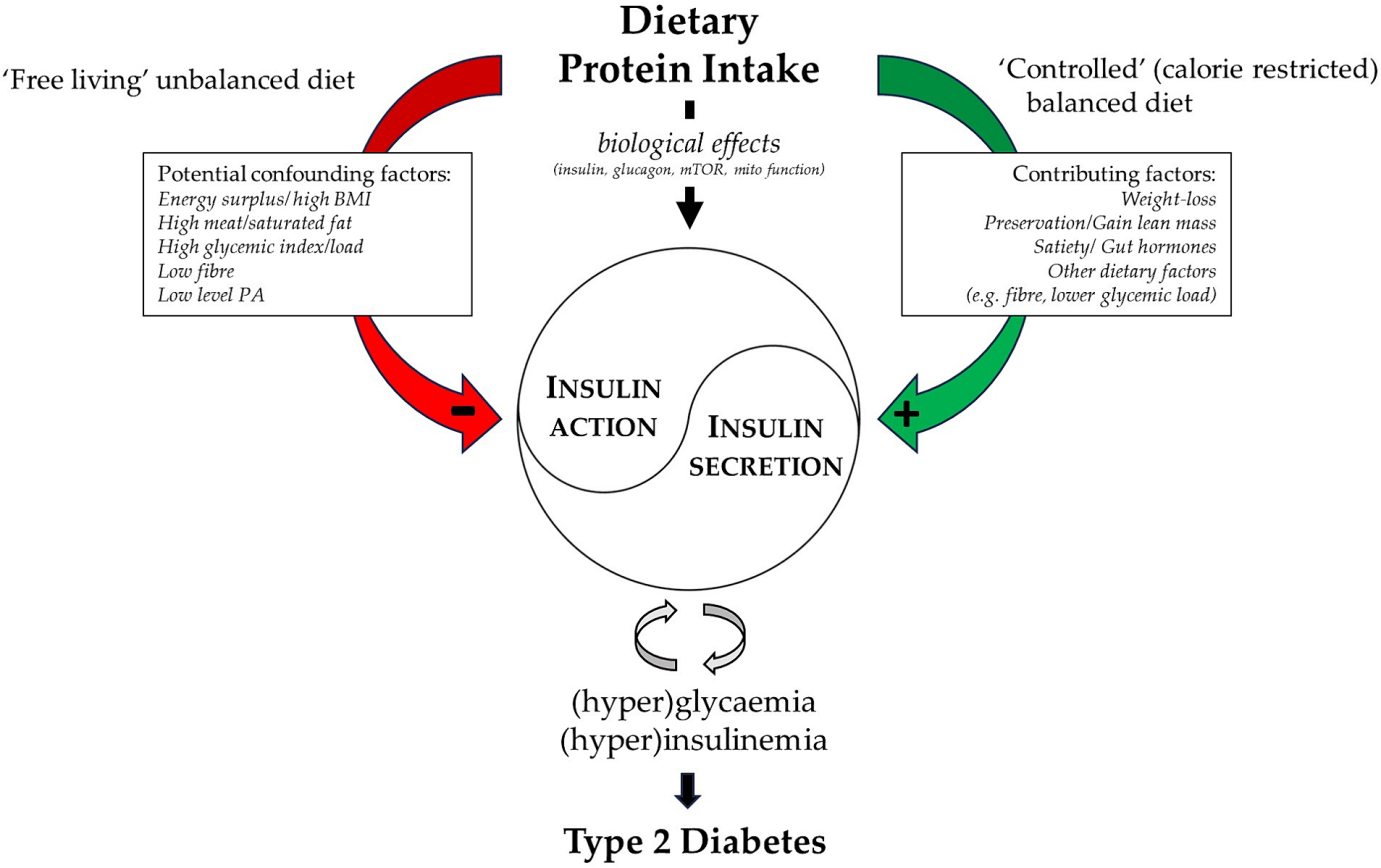
Visual summary of the different links between dietary protein intake and type 2 diabetes. A high protein intake associates with several potential confounders in “free living” (observational) conditions and is linked to an impaired insulin and glucose regulation and increased diabetes risk. While in controlled energy restricted (intervention) conditions several factors contribute to the beneficial link between high protein diets and an improved glucose metabolism. With central the biological effects that could either facilitate glycaemic control (i.e., insulin secretion), or have potentially detrimental effects (i.e., hyperglucagonemia, mTOR activation and/or impaired mitochondrial function).

Protein and AA play a crucial role in human physiology and metabolism. Low protein diets and diets with a low or inadequate protein and/or essential AA uptake are associated with severe health risks. The effect of high protein diets and high protein consumption on cardiometabolic disease, in particular type 2 diabetes, is much more complex and multifaceted (Figure 1).

Proteins and AA can have multiple biological effects. Acutely, the insulin secretory properties of AA can improve glycaemic control in patients with diabetes, while its effect on glucagon secretion has the potency to impair glucose regulation and is associated with insulin resistance. Protein intake, in particular when combined with exercise, stimulates protein synthesis and can improve body composition during weight-loss with beneficial health effects, including an improved glucose metabolism. But BCAA dysmetabolism and as a consequence elevated BCAA levels and its metabolites can on the long-term have detrimental effect on insulin action and mitochondrial function facilitating the development of insulin resistance and an impaired glucose regulation.

Under ‘free living’ condition a high protein consumption is in general associated with an unbalanced ‘western’ diet, with an excess of calories and increased BMI, a high meat and saturated fat intake, a high glycaemic load and low fiber intake. This results in observational studies identifying a high protein consumption as a risk factor for type 2 diabetes. In controlled settings with a (calorie-restricted) balanced diet a high protein intake, together with a low glycaemic load, high fiber intake and weight loss with preservation of lean mass can improve insulin resistance and metabolic control (Figure 1).

The conclusions from our review 10 years ago (11) remain valid: high protein, non-energy restricted diets seem not warranted to reduce insulin resistance or prevent diabetes. Long-term high-protein intake maybe even having deleterious effects, when diets are unbalanced. High-protein energy restricted diets to support weight loss and lean muscle accretion can be considered to improve metabolic control in those with—or at risk for—type 2 diabetes. To improve glycaemic control other dietary changes like restricting energy intake, reducing total and saturated fat intake and increasing fiber intake should however be of higher priority.

## Author contributions

MM: Writing – original draft, Writing – review & editing.
